# The basis and advances in clinical application of boron neutron capture therapy

**DOI:** 10.1186/s13014-021-01939-7

**Published:** 2021-11-07

**Authors:** Huifang He, Jiyuan Li, Ping Jiang, Suqing Tian, Hao Wang, Ruitai Fan, Junqi Liu, Yuyan Yang, Zhibo Liu, Junjie Wang

**Affiliations:** 1 Department of Radiotherapy, Peking University International Hospital, Beijing, China; 2 College of Chemistry and Molecular Engineering, Peking University, Beijing, 100871 China; 3 Department of Radiotherapy, Peking University 3rd Hospital, Beijing, 100191 China; 4 Department of Radiation Oncology, The First Affiliated Hospital of Zhengzhou University, Zhengzhou, Henan China

**Keywords:** Boron neutron capture therapy, Boron carriers, Neutron source, Tumor

## Abstract

Boron neutron capture therapy (BNCT) was first proposed as early as 1936, and research on BNCT has progressed relatively slowly but steadily. BNCT is a potentially useful tool for cancer treatment that selectively damages cancer cells while sparing normal tissue. BNCT is based on the nuclear reaction that occurs when ^10^B capture low-energy thermal neutrons to yield high-linear energy transfer (LET) α particles and recoiling ^7^Li nuclei. A large number of ^10^B atoms have to be localized within the tumor cells for BNCT to be effective, and an adequate number of thermal neutrons need to be absorbed by the ^10^B atoms to generate lethal ^10^B (n, α)^7^Li reactions. Effective boron neutron capture therapy cannot be achieved without appropriate boron carriers. Improvement in boron delivery and the development of the best dosing paradigms for both boronophenylalanine (BPA) and sodium borocaptate (BSH) are of major importance, yet these still have not been optimized. Here, we present a review of this treatment modality from the perspectives of radiation oncology, biology, and physics. This manuscript provides a brief introduction of the mechanism of cancer-cell-selective killing by BNCT, radiobiological factors, and progress in the development of boron carriers and neutron sources as well as the results of clinical study.

## Introduction

In oncologic therapeutic areas, scientists continue to explore and develop innovative therapies that are more effective and less toxic in order to improve the local tumor control rates, patient survival, and life quality. Boron neutron capture therapy (BNCT) was proposed decades ago as an innovative radiotherapy approach and, in theory, could be an ideal treatment for many types of cancer. Treatment begins with an injection of boron containing drugs; the drug and cancer cells have a strong affinity and quickly gather within the tumor cells, and rarely in normal tissues. Then, thermal neutron irradiation was carried out to the tumor site of patients. When thermal neutrons were captured by ^10^B in the tumor cells, fission occurred, producing high lethal α particle and recoiling ^7^Li nuclei that can precisely “kill” the tumor cells. In the first half of this paper, the mechanism and biological factors of killing tumor cells by BNCT were introduced. In the latter section, boron carriers, neutron sources, and clinical results are discussed.

## Discussion

### Mechanism of selective killing of tumor cells with BNCT

**Fig. 1:**
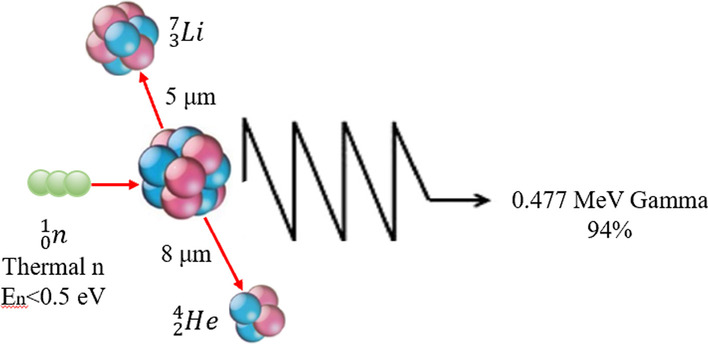
Boron neutron capture reaction

**Fig. 2:**
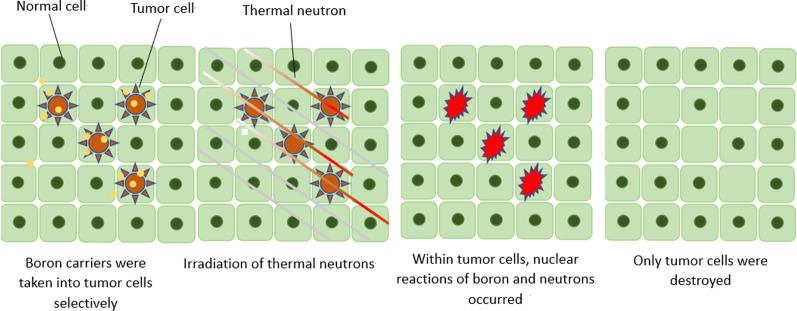
The mechanism of selective killing of tumor cells with boron neutron capture therapy

BNCT is a fission reaction based on boron neutron capture for tumor treatment [1, 2]. Non-radioactive isotope ^10^B atoms fission into an α (^4^He) particle and a recoil lithium nucleus (^7^Li) via the absorption of low-energy (< 0.5 eV) neutrons (thermal neutrons). These particles release energy over a short- range (< 10 μm). The size of a single cell is about 10 μm; thus, the boron neutron capture reaction occurs in a single cell (shown in Fig. 1). Theoretically, ^10^B can be selectively aggregated in malignant tumor cells; thus, BNCT selectively kills tumor cells with two heavy particles (^4^He and ^7^Li), while it protects normal tissue from harm (shown in Fig. 2).

### Basic radiobiological factors of BNCT

During BNCT, there are three direct ionization energies with different linear energy transfer (LET) characteristics that are transmitted to the tumor and normal tissues, as follows: (a) High-LET α particles (^4^He) and ^7^Li ions produced by the neutron capture reaction of boron: ^10^B + ^1^n = ^7^Li + ^4^He(α) + 2.79 MeV; (b) The low-LET gamma rays contained in the neutron beam and produced by the hydrogen neutron capture reaction: ^1^H + ^1^n = ^2^H + γ + 2.2 MeV; (c) High-LET protons produced by fast neutron scattering and thermal neutron capture by nitrogen atoms: ^14^N + ^1^n = ^14^C + ^1^p + 580 keV. Even if the physical dose is the same, the biological effects of high-LET and low-LET particles differ. In the case of the same physical dose, the higher-LET particles produce stronger biological effects than the lower-LET particles owing to the higher ionization density along the trajectories. This is commonly referred to as the relative biological effect and is equal to the ratio of the absorbed dose of a reference radiation source (such as X-rays) to the absorbed dose of other radiation sources that produce the same biological effect.

For a more accurate assessment of the biological effects of BNCT, it is necessary to determine the compound biological effects (CBE)—the coefficients used to convert neutron and boron doses into equivalent X-ray doses. The value of the composite biological effect can be obtained from the dose ratio corresponding to the biological effect of the BNCT of the photon beam in the experiment, where a and b are the absorbed dose of the standard radiation of the photon and any BNCT respectively. The value of the composite biological effect can be obtained as per the dose ratio corresponding to the biological effect of the BNCT of the photon beam in the experiment (D^ref^/D^*^, where D^ref^ and D^*^ are the absorbed dose of the standard radiation of the photon and any BNCT respectively). BNCT cannot be used to treat cancer in any tissue or organ where the CBE value has not been determined [3]; thus, the experimental evaluation of CBE value is an urgent issue for advancing the development of BNCT. In addition, a fixed value is currently being used for the provisional CBE value of tumors in BNCT clinical studies; however, this confuses the relationship between the estimated bioequivalent dose and the actual therapeutic effect.

### Boron carriers

BNCT kills tumor cells mainly based on the following two points: ^10^B and thermal neutrons. The high concentration and selective delivery of ^10^B to the tumor tissues are the most important factors for the treatment of tumors with boron neutron capture [4, 5]. In order for BNCT to achieve the lethal destruction of tumor cells, the following three important factors need to be considered in the development of boron carriers: (1) The concentration range of ^10^B in the tumor tissues should be 20–35 μg ^10^B/g; (2) The tumor/normal tissue 10B concentration ratio and the tumor/blood 10B concentration ratio were greater than 3–5; and (3) Toxicity should be sufficiently low [6]. However, currently, there is no boron carrier that meets all the above requirements.

**Fig. 3:**
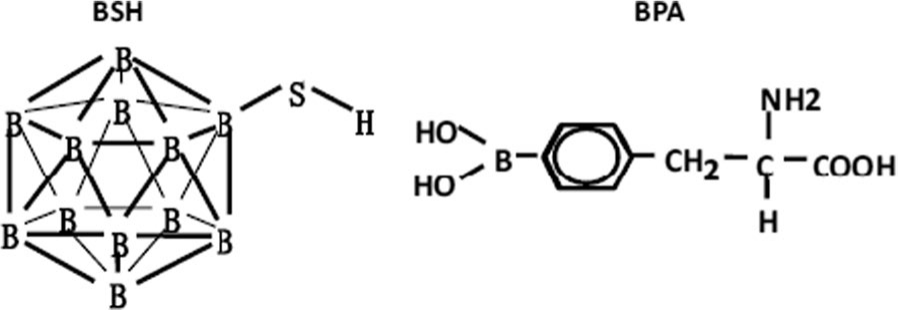
Boron carriers used in clinical studies in boron neutron capture therapy

In the 1950s, the development of neutron capture therapy was one of the first development goals of the medical community in search of better cancer treatments. In the 1950s and early 1960s, BNCT development was in its infancy, with preliminary clinical trials using borax, boric acid, and their derivatives as boron carriers [7, 8]. To evaluate the effect of BNCT on brain tumors. Farr et al. reported the first clinical trial of BNCT in patients with glioblastoma multiforme using borax as a boron carrier. The blood–brain barrier is a major barrier to the dispersion of boron carriers in the brain. The blood–brain barrier was damaged in tumors, and ^10^B dispersed faster in tumors than in normal brain tissue. Before the boron concentration in the brain equilibrates, there is a short and uncontrollable period of time when T/N levels are high and neutron irradiation is barely possible. Obviously, the first generation of boron carrier has serious defects: (1) It does not have tumor specificity, and cannot selectively transport ^10^B to tumor cells, and normal tissues are seriously damaged after neutron irradiation; (2) Rapid removal of boron from tumor cells and insufficient concentration of boron in the tumor. Therefore, the first clinical trials of BNCT failed [9, 10] and it is urgent to develop new, more efficient boron carriers. The first generation of boron agents to carry the main shortcoming is the lack of tumor specificity, this led directly to the ideal BNCT efficacy and serious side effects. 50-60 s of the twentieth century, the researchers devoted to the synthesis and evaluation of the various new type of boron compounds, to develop to selectively target tumor boron carrying agent. Boronophenylalanine (BPA) and borocaptate sodium (BSH) (shown in Fig. 3), emerged from these two substances and gradually developed into the second generation of BNCT boron carriers. The design of BPA is inspired by the metabolic pathway of amino acids. Due to its similar structural characteristics to tyrosine, BPA participates in the synthesis of specific proteins, thus selectively accumulating in tumor growth related proteins during the rapid proliferation of tumor cells, thus achieving the specific binding of BPA to the tumor. Snyder et al. [11] reported the synthesis method of BPA. BPA-BNCT was originally used to treat cutaneous melanoma because BPA is involved in melanin synthesis and preferentially ingested by melanoma cells [12]. BPA is also an effective boron carrier for brain tumors because there are few proliferating cells in the brain other than malignant tumors. Yoshino et al. [13] combined BPA with fructose (BPA-F) to significantly increase the water solubility of BPA under physiological pH and further improve the ability of ^10^B delivery to tumor cells. Coderre et al. [14] demonstrated that BPA can carry a therapeutic concentration of ^10^B to target a variety of tumor types, including 9L rat glioma. This has encouraged researchers to promote BNCT clinical trials of high-grade glioma using BPA-F as boron carrier [15–17], and gradually extended to the treatment of other extracranial tumors, such as head and neck cancer. BSH is developed with the development of carborane chemistry, which usually contains more than 10 boron atoms. In 1967, Solow et al. [18] synthesized [B_12_H_12_]^2−^ mercapto derivative sodium salt Na_2_[^10^B_12_H_11_SH], namely BSH, and proved that BSH has brain tumor specificity through experiments. Due to the abundance of boron atoms, BSH is favored by BNCT clinical researchers, so a large number of clinical trials of BSH-BNCT in advanced glioma have been conducted in Japan and other regions [19, 20].

Due to the different mechanism of action [21], BPA mainly targets proliferating tumor cells and produces higher boron concentration in tumor cells, thus BPA-BNCT has greater tumor damage and lower side effects than BSH-BNCT [22]. The combination of BPA and BSH can obtain a more uniform distribution of tumor boron and achieve better BNCT efficacy. Improved administration of BPA and BSH, such as intravenous mannitol hypertonic solution [23, 24], focused ultrasound [25], or direct intratoma administration via convection-enhanced delivery, significantly increased uptake of BPA and BSH in brain tumors and improved the efficacy of BNCT. With the gradual maturation of positron emission tomography (PET) technology, ^18^F-labeled BPA can visually present the distribution of ^10^B in the human body through PET imaging, providing constructive guidance for the formulation and implementation of BNCT treatment plan [26–28]. After decades of development, only BPA and BSH are currently used in clinical practice, in which BPA is the preferred boron carrier for clinical BNCT in patients with high-grade glioma and recurrent head and neck tumors. Although the second generation of boron carriers is not perfect, they have extended the lives of millions of patients and represent an important milestone in the development of boron carriers.

The second generation of boron carriers has made remarkable achievements in clinical application. However, due to the defects in molecular characteristics, BPA and BSH are not specific enough to meet the ideal standard of boron carriers. In order to make BNCT a more effective cancer treatment, more researchers have invested in the research and development of boron carriers with the hope of designing and developing new boron carriers with higher tumor specificity. Based on the standard of the best boron carrier, the development of various types of boron carriers is flourishing, and the development of the third generation of boron carriers shows vigorous vitality. In summary, it can be divided into two categories: boron containing small molecules and boron containing nano-drugs.

Boron-containing amino acid derivatives as a promising type of small-molecule boron carriers can achieve tumor specificity through amino acid metabolism pathway and have potential as new boron carriers. BPA is a typical natural amino acid derivative, which can be selectively accumulated in tumors by amino acid transporters rich in tumors such as LAT1, and has been widely used in clinical BNCT. A variety of new boron carriers have been derived from BPA and ^18^F-BPA. Due to the effect of anti-transport mechanism, BPA is easy to be excreted by tumor cells and the retention time is shorter. Recently, Nomoto et al. [29] combined BPA with polyvinyl alcohol (PVA-BPA) through boric acid esterification to internalize BPA into tumor cells through endocytogenesis, thereby enhancing tumor uptake and reducing effervescent, prolongated retention time, and significantly enhancing the efficacy of BNCT. Considering that the metabolic instability of ^18^F-BPA under high H_2_O_2_ concentration in the tumor would affect its boron carrying capacity and imaging reality, Li et al. [30] synthesized a novel Boronated-tyrosine FBY. ^18^F-FBY is immune to H_2_O_2_-mediated boron removal reaction, has strong metabolic stability, and shows high tumor specificity. It can significantly prolong the survival of B16-F10 tumor-bearing mice under neutron irradiation and is a potential boron carrier for PET imaging.

As for the nano carrier side, boron containing micelles were intensively studied recently. Block copolymers can self-assemble into spherical core–shell polymer micelles in water. They are simple to prepare, have high biocompatibility, good stability, and strong water solubility. The copolymerization with PEG can effectively prolong the circulation time in blood, and the hydrophobic core can load insoluble drugs to achieve solubilization. As a practical nano-carrier, it is widely used in drug imaging and targeted delivery and plays an important role in the diagnosis and treatment of cancer.

Sumitani et al. [31, 32] introduced the PEG end into the aldehyde group, PEG-PLA containing boron was synthesized by cross-linking PEG-PLA with methyl acryloyl at the PLA end and polymerizable carborane. This cross-linking loading form avoided carborane leakage from the micelles, prolonged blood circulation time, and showed high tumor uptake. Boron-containing PEGPLA micelles are simple to prepare and can effectively inhibit tumor growth in tumor-bearing mice when applied to BNCT, showing an important clinical application prospect [33].

Compared with PEG-PLA, PLGA has stronger hydrophilicity and better degradation performance and is also a very useful micellar drug carrier [34, 35]. Shi et al. [36] used methoxy-polyethylene glycol—polylactide—ethyl ester (mPEG-PLGA) micelles to coat boronaedporphyrin TBPP. The nano-drug BPN containing boron was prepared. Micellar coating not only isolates TBPP from blood cells and reduces drug toxicity, but also enhances tumor specificity and overcomes the shortcomings of traditional boron-containing porphyrins. The pharmacokinetics of BPN can also be studied through fluorescence imaging and PET imaging to facilitate the development of treatment regimens. BPN also successfully delivered boron to the nucleus, and BNCT almost completely inhibited melanoma growth in B16-F10 tumor-bearing mice, making it a promising tracer of boron carriers.

### Neutron source for BNCT

#### Reactor neutron source

According to their energy, the neutrons produced in the nuclear reactors can be classified as thermal neutrons (E_n_ < 0.5 eV), epithermal neutrons (0.5 eV < E_n_ < 10 keV), and fast neutrons (E_n_ > 10 keV). Thermal neutron is the most important neutron source in BNCT, that can participate in the boron neutron capture reaction. All the reported reactions in patients who have received boron neutron capture therapy have been carried out at different nuclear reactors in multiple countries. However, the prospect is not optimistic because of the high cost, large footprint, difficulty in retrofitting, and high cost of operation and maintenance. Many reactors are no longer open, some have ceased BNCT activity, and some are at risk of closure; thus, only a few can be used for BNCT. However, some new designs regarding reactor neutron sources have been proposed or are under construction, such as that of a low-power reactor located in the suburbs of Beijing, China; this reactor involves a low cost and high degree of safety and is particularly suitable for hospital internal use, is specially designed for neutron capture therapy [37, 38]; however, this reactor may not ever work in any other country. At present, the only hope for large-scale clinical trials is the use of an accelerator-based neutron source to produce qualified neutron beams that can be extended to general hospitals for BNCT treatment of tumors.

#### Accelerator-based BNCT neutron source (AB-BNCT)

**Fig. 4:**
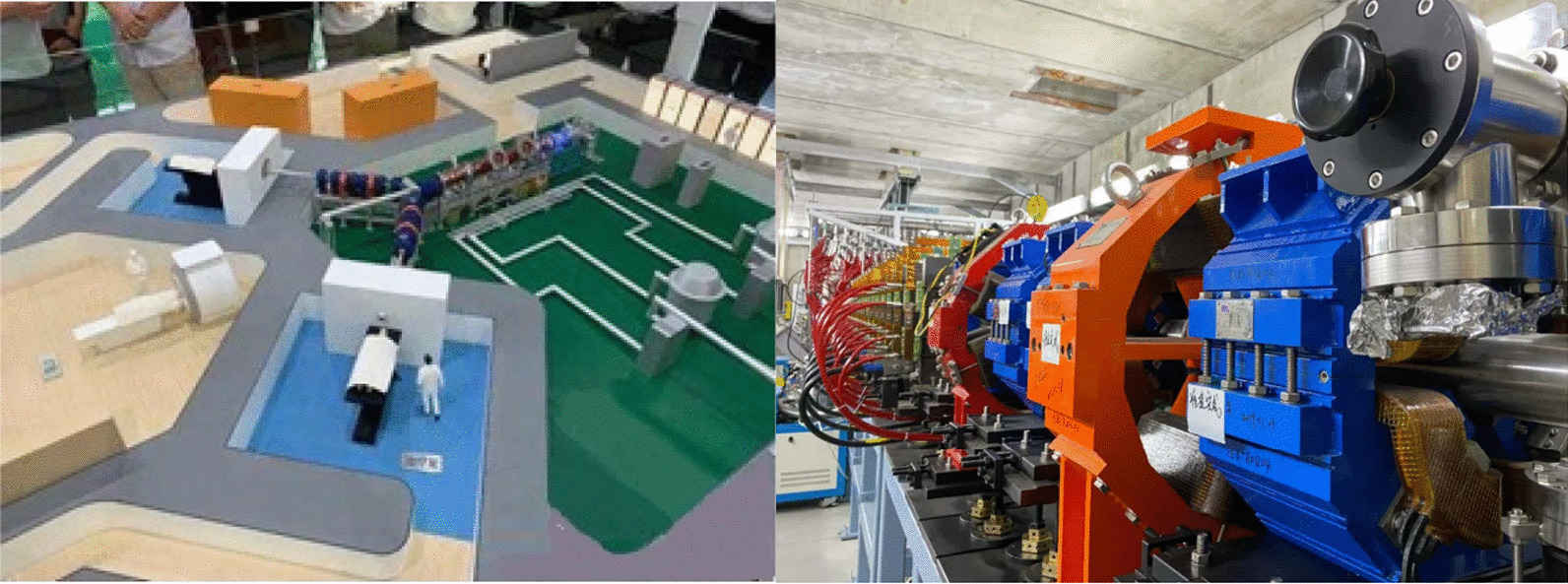
China's first self-developed accelerator-based BNCT device

The concept of accelerators for BNCT was first proposed > more than 30 y ago. Generally, protons or deuterium ions are first accelerated by an accelerator and then bombarded with lithium or beryllium metal targets to produce neutrons via fission. For AB-BNCT, the most promising nuclear reaction is that where ^7^Li targets are bombarded by 2.5 MeV protons, producing neutrons with maximum and average neutron energies of 0.8 MeV and 0.4 MeV, respectively, lower than those produced by a reactor. This is because the thickness of the moderator needed to reduce the energy of the neutrons from fast neutrons to epithermal neutrons is smaller than that of a reactor. As per the requirements of the International Atomic Energy Agency, the flux of the neutron source for BNCT must be > 10^9^ n/cm^2^/s. Before 2014, the neutron sources used for BNCT were special thermal or epithermal neutrons produced by nuclear reactors; thereafter, three Japanese industries Sumitomo Heavy, Hitachi, and Mitsubishi, as well as an American neutron treatment company (Danforth, MA, USA), manufactured an AB-BNCT neutron source that could be installed in hospitals and produce the epithermal neutron beams. At present, many different types of neutron source accelerators, such as low-energy linac, high-energy cyclotron, high-energy linac, and high-energy synchrotron are being considered for BNCT. AB-BNCT has many advantages over reactor neutron sources, as follows: (a) Accelerators can be easily shut down when no neutron source is needed, and reactors have large amounts of permanent radioactive material left over; (b) Accelerators have an easier licensing process than reactors; (c) The installation and maintenance of accelerators is easier than that of reactors; (d) The AB-BNCT system is much cheaper than the cost of installing a reactor system in or near a hospital; (e) The radiotherapy department of the hospital has many years of experience in using accelerators; (f) It is very important that the mass of the neutron source produced by the accelerator is much higher than that produced by the reactor. Several clinical AB-BNCT development projects are ongoing; the most advanced cyclotron has been developed by Sumitomo Heavy Industries (SHI). In 2009, they successfully developed a cyclotron-based neutron source, in collaboration with Kyoto University [39]. In addition, in 2012, the Institute of Integrated Radiation Research at Kyoto University started the world’s first accelerator-based BNCT clinical trial using this device [40]. In 2020, Shi and Stella Pharmaceuticals acquired the right to manufacture and sell the accelerator-based BNCT equipment and dose calculation program from the Ministry of Health, Labor, and Welfare of Japan for the first time. Recently, China has also made considerable progress in AB-BNCT research and development. In August 2020, China’s first independently developed AB-BNCT experimental device was successfully developed in Dongguan (shown in Fig. 4), and relevant experiments have been initiated; clinical trials are expected to be performed in the near future.

### Clinical studies of BNCT

The boron carriers selectively aggregate in the tumor cells and are then irradiated by an ultra-thermal neutron beam, resulting in a large dose gradient between the tumors and normal cells with the selective destruction of tumor cells. Compared with photon therapy and charged particle radiotherapy, BNCT is theoretically a promising tool for cancer treatment due to the highly focused radiation on the tumor, without irritating the normal tissue, as the compound targeted the tumor and only works when the neutron beam was given. However, BNCT is in this infancy stage and only exploratory studies were currently reported without directly comparison with other radiotherapy modality.

Currently, emerging BNCT is becoming a promising tool for cancer treatment. BNCT clinical studies have been carried out in Japan [41], Finland [42], the United States of America [43] Taiwan [44], Netherlands [45], Germany [46], Italy [47], Argentina [48], the Czech Republic [49], and Sweden [50]. At present, this treatment modality is mainly being used in the treatment of malignant glioma [51–54] and cutaneous melanoma; recently, it has been most commonly used for recurrent head and neck tumors for patients in whom conventional treatment has failed [55–62].

#### Malignant glioma

A clinical study of accelerator-based BNCT for malignant glioma in Japan showed that the median survival time (MST) was 10.8–27.1 mon [63]. The results were comparable to those of conventional radiotherapy [52]. Miyatake et al. [53, 64, 65] reported that in a cohort of 22 patients with recurrent malignant gliomas, there was a significant prolongation in the average survival after BNCT (9.1 mon) than that (4.4 mon) for 28 patients who had received other types of salvage therapy following recurrence. The clinical data show that BNCT was effective as adjuvant treatment for newly diagnosed glioblastoma multiforme after surgical resection. Kawabata et al. reported that the MST of patients with newly diagnosed glioblastoma multiforme who underwent BNCT after surgical resection with (n = 11) or without (n = 10) subsequent additional fractionated radiotherapy boost was 23.5 mon and 14.1 mon, respectively [66]. Although there was little difference in the survival time between BNCT and “standard” conventional radiotherapy with temozolomide, the best survival data of the recent BNCT (MST 19–26 months) appeared to be comparable to the best results of recent studies of high-dose radiotherapies.

#### Recurrent tumors of the head and neck region

The second largest group of patients receiving BNCT is those with recurrent head and neck tumors who have undergone surgery, chemotherapy, and photon radiation with doses that reached normal tissue tolerance levels in the absence of other treatment options. Head and neck cancers (HNCs) account for about 10% of all cancers, and about 90% of them are squamous cell carcinomas (SCCs). Recurrent HNSCCs show good response to BNCT [64]. As per the Response Evaluation Criteria for solid tumors, the reports of response rates ranged from 61 to 100%. Although the number of patients with HNSCC treated with BNCT is relatively small, some cases have shown very impressive clinical results. Fuwa N et al. reported a mean survival duration of 33.6 mon with a high response rate of 85% in 26 patients with recurrent HNCs (19 SCCs, 4 salivary gland carcinomas, and 3 sarcomas) who had recurrence following standard treatment and were subjected to BNCT [67]. Wang et al. [68] reported that almost all the patients experienced some degree of pain relief and improvement in the quality of life at least shortly after BNCT for 12 patients with recurrent HNCs. The best response was complete response in four patients, partial response in three, stable disease in two, and progression of disease in three. In the treatment of refractory head and neck tumors, a single day of BNCT therapy resulted in a high tumor regression rate, suggesting that BNCT is a viable option for palliative treatment [45].

#### Cutaneous melanoma

Cutaneous melanomas are malignant tumors arising from melanocytes, that is, pigmented cells, and represent the most common type for melanomas. Although surgical excision is considered the most effective treatment for cutaneous melanoma and few patients are treated with BNCT because of the high tolerance to irradiation for melanoma, some interesting clinical studies have been performed. Between 2003 and 2014, eight patients with cutaneous melanoma were treated with BNCT by Hiratsuka et al. in Japan [69]. All the lesions gradually subsided within 1 y. The complete and partial response rates were 75% (6/8) and 25% (2/8), respectively, and no complications have been reported, suggesting that BNCT may be a promising treatment modality for cutaneous melanomas. As reported by Fukuda et al. [49], 32 patients with cutaneous melanoma were treated with BNCT using BPA as the boron carriers. The complete response rate was as high as 78%. The most common complications were edema and skin erosion at the radiation site.

## Conclusion

Thus, it is clear from this review that BNCT is a treatment method that combined nuclear physics, chemistry, biology, medicine, and other disciplines. The concept of BNCT was proposed in 1936 and has brought new hope in the field of cancer treatment. After decades of clinical research and development, BNCT has become a highly effective technology for cancer treatment. There is an urgent need to optimize the use of BPA and BSH alone or in combination or to develop new boron carriers that would improve tumor cell uptake and cell micro distribution, especially for different subpopulations of tumor cells. In addition, it is necessary to conduct randomized clinical trials for evaluating the safety and efficacy of BNCT. Although BNCT is not widely available at present, BNCT might potentially become a milestone in the field of oncology therapy in the near future.

## Data Availability

Not applicable.

## Abbreviations

BNCT: Boron neutron capture therapy
LET: Linear energy transfer
BPA: Boronophenylalanine
BSH: Sodium borocaptate
CBE: Compound biological effects
PVA: Polyvinyl alcohol
PET: Positron emission tomography
mPEG-PLGA: Methoxy-polyethylene glycol-polylactide-ethyl ester
AB-BNCT: Accelerator-based BNCT
y: Year
MST: Median survival time
HNCs: Head and neck cancers
SCCs: Squamous cell carcinomas

## References

[1] Locher GL (1936). Biological effects and therapeutic possibilities of neutrons. Am J Roentgenol Radium Ther.

[2] Coderre JA, Morris GM (1998). The radiation biology of boron neutron capture therapy. Radiat Res.

[3] Ono K (2016). An analysis of the structure of the compound biological effectiveness factor. J Radiat Res.

[4] Soloway AH, Tjarks W, Barnum BA (1998). The chemistry of neutron capture therapy. Chem Rev.

[5] Hawthorne MF (1993). The role of chemistry in the development of boron neutron capture therapy of cancer. Angew Chem Int Ed Engl.

[6] Barth RF, Soloway AH, Fairchild RG (1992). Boron neutron capture therapy for cancer, realities and prospects. Cancer.

[7] Farr LE, Sweet WH, Robertson JS (1954). Neutron capture therapy with boron in the treatment of glioblastoma multiforme. Am J Roentgenol Radium Ther Nucl Med.

[8] Godwin JT, Farr LE, Sweet WH (1955). Pathological study of eight patients with glioblastoma multiforme treated by neutron-capture therapy using boron 10. Cancer.

[9] Sweet WH, Javid M (1952). The possible use of neutron-capturing isotopes such as boron 10 in the treatment of neoplasms. I Intracranial tumors. J Neurosurg.

[10] Asbury AK, Ojemann RG, Nielsen SL (1972). Neuropathologic study of fourteen cases of malignant brain tumor treated by boron-10 slow neutron capture radiation. J Neuropathol Exp Neurol.

[11] Snyder HR, Reedy AJ, Lennarj WJ (1958). Synthesis of aromatic boronic acids. Aldehyde boronic acids and a boronic acid analog of tyrosine. J Am Chem Soc.

[12] Mishima Y, Ichihashi M, Hatta S (1989). First human clinical trial of melanoma neutron capture. Diagnosis and therapy. Strahlenther Onkol.

[13] Yoshino K, Suzuki A, Mori Y (1989). Improvement of solubility of p-boronophenylalanine by complex formation with monosaccharides. Strahlenther Onkol.

[14] Coderre JA, Glass JD, Fairchild RG (1990). Selective delivery of boron by the melanin precursor analogue p-boronophenylalanine to tumors other than melanoma. Cancer Res.

[15] Coderre JA, Elowitz EH, Chadha M (1997). Boron neutron capture therapy for glioblastoma multiforme using p-boronophenylalanine and epithermal neutrons: trial design and early clinical results. J Neurooncol.

[16] Sköld K, H-Stenstam B, Diaz AZ (2010). Boron neutron capture therapy for glioblastoma multiforme: advantage of prolonged infusion of BPA-f. Acta Neurol Scandin..

[17] Kankaanranta L, Seppälä T, Koivunoro H (2011). L-boronophenylalanine-mediated boron neutron capture therapy for malignant glioma progressing after external beam radiation therapy: a Phase I study. Int J Radiat Oncol.

[18] Soloway AH, Hatanaka H, Davis MA (1967). Penetration of brain and brain tumor. VII. Tumor-binding sulfhydryl boron compounds. J Med Chem.

[19] Hatanaka H, Nakagawa Y (1994). Clinical results of long-surviving brain tumor patients who underwent boron neutron capture therapy. Int J Radiat Oncol.

[20] Nakagawa Y, Pooh K, Kobayashi T (2003). Clinical review of the Japanese experience with boron neutron capture therapy and a proposed strategy using epithermal neutron beams. J Neuro-Oncol.

[21] Ono K, Masunaga SI, Kinashi Y (1996). Radiobiological evidence suggesting heterogeneous microdistribution of boron compounds in tumors: its relation to quiescent cell population and tumor cure in neutron capture therapy. Int J Radiat Oncol.

[22] Miyatake SI, Kawabata S, Kajimoto Y (2005). Modified boron neutron capture therapy for malignant gliomas performed using epithermal neutron and two boron compounds with different accumulation mechanisms: an efficacy study based on findings on neuroimages. J Neurosurg.

[23] Barth RF, Yang W, Rotaru JH (1997). Boron neutron capture therapy of brain tumors: enhanced survival following intracarotid injection of either sodium borocaptate or boronophenylalanine with or without blood-brain barrier disruption. Cancer Res.

[24] Yang W, Barth RF, Rotaru JH (1997). Enhanced survival of glioma bearing rats following boron neutron capture therapy with blood-brain barrier disruption and intracarotid injection of boronophenylalanine. J Neurooncol.

[25] Burgess A, Shah K, Hough O (2015). Focused ultrasound-mediated drug delivery through the blood-brain barrier. Expert Rev Neurother.

[26] Imahori Y, Ueda S, Ohmori Y (1998). Focused ultrasound-mediated drug delivery through the blood-brain barrier. J Nucl Med.

[27] Kato I, Ono K, Sakurai Y (2004). Effectiveness of BNCT for recurrent head and neck malignancies. Appl Radiat Isot.

[28] Aihara T, Hiratsuka J, Morita N (2006). First clinical case of boron neutron capture therapy for head and neck malignancies using 18F-BPA PET. Head Neck.

[29] Nomoto T, Inoue Y, Yao Y (2020). Poly(vinyl alcohol) boosting therapeutic potential of p-boronophenylalanine in neutron capture therapy by modulating metabolism. Sci Adv.

[30] Li J, Shi Y, Zhang Z (2019). A metabolically stable boron-derived tyrosine serves as a theranostic agent for positron emission tomography guided boron neutron capture therapy. Bioconj Chem.

[31] Sumitani S, Oishi M, Nagasaki Y (2011). Carborane confined nanoparticles for boron neutron capture therapy: improved stability, blood circulation time and tumor accumulation. Reactive Funct Polyms.

[32] Sumitani S, Nagasaki Y (2012). Boron neutron capture therapy assisted by boron-conjugated nanoparticles. Polym J.

[33] Sumitani S, Oish M, Yaguchi T (2012). Pharmacokinetics of core-polymerized, boron-conjugated micelles designed for boron neutron capture therapy for cancer. Biomaterials.

[34] Mir M, Ahmed N, Rehman A (2017). Recent applications of PLGA based nanostructures in drug delivery. Colloids Surfs B Biointerfaces.

[35] Zhang K, Tang X, Zhang J (2014). PEG-PLGA copolymers: their structure and structure-influenced drug delivery applications. J Control Release.

[36] Shi Y, Li J, Zhang Z (2018). Tracing boron with fluorescence and positron emission tomography imaging of boronated porphyrin nanocomplex for imaging-guided boron neutron capture therapy. ACS Appl Mater Interfaces.

[37] Zhou Y, Gao Z, Li Y, Guo C, Liu X. Design and construction of the inhospital neutron irradiator-1 (HNI). In: Nakagawa Y, Kobayashi T, Fukuda H, editors. Proceedings of the 12th ICNCT—advances in neutron capture therapy 2006; October 9–13, Takamatsu, Japan. P. 557–60.

[38] Li Y, Xia P, Wang X, Kong F, Huang Q. Start-up of the first in hospital Neutron Irradiator (IHNI1) & presentation of the BNCT development status in China. In: Liberman S, Kreiner AJ, Casal MR, Menendez P, Schwint A, Dragosa A, Cruz GS, editors. Proceedings of the 14th international congress on neutron capture therapy, new challenges in neutron capture therapy. 2010; October 25–29. Buenos Aires, Argentina. p. 371–4.

[39] Tanaka H, Sakurai Y, Suzuki M, Masunaga S, Kinashi Y, Kashino G (2009). Characteristics comparison between a cyclotron-based neutron source and KUR-HWNIF for boron neutron capture therapy. Nucl Instrum Methods Phys Res.

[40] Hatanaka H, Karin ABMF, Laws E (1991). Boron neutron capture therapy for brain tumors. Glioma.

[41] Ono K, Ueda S, Oda Y, Nakagawa Y, Miyatake S, Osawa M, Kobayashi T, Larsson B, Crawford J, Weinreich R (1997). Boron neutron capture therapy for malignant glioma at Kyoto University reactor. Advances in neutron capture therapy.

[42] Joensuu H, Kankaanranta L, Seppälä T (2003). Boron neutron capture therapy of brain tumors: clinical trials at the Finnish facility using boronopheny lalanine. J Neuro-Oncol.

[43] Chanana AD, Capala J, Chadha M (1999). Boron neutron capture therapy for glioblastoma multiforme: interim results from the phase I/II dose-escalation studies. Neurosurgery.

[44] Liu YW, Huang T, Jiang S, Liu H (2004). Renovation of epithermal neutron beam for BNCT at THOR. Appl Radiat Isot.

[45] Sauerwein W, Zurlo A, Group EBNCT (2002). The EORTC boron neutron capture therapy (BNCT) group: achievements and future projects. Eur J Cancer.

[46] Wittig A, Hideghety K, Paquis P, et al. Current clinical results of the EORTC-study 11961. In: Research and development in neutron capture therapy. 2002. p. 1117–22.

[47] Pinelli T, Zonta A, Altieri S, et al. TAOrMINA: from the first idea to the application to the human liver. In: Research and development in neutron capture therapy. 2002. p. 1065–72.

[48] González S, Bonomi M, Santa Cruz G (2004). First BNCT treatment of a skin melanoma in Argentina: dosimetric analysis and clinical outcome. Appl Radiat Isot.

[49] Dbaly V, Tovarys F, Honova H (2003). Contemporary state of neutron capture therapy in the Czech Republic (part 2). Ceska a Slovenska Neurologie a Neurochirurgie.

[50] Capala J, Britta H, Sköld K (2003). Boron neutron capture therapy for glioblastoma multiforme: clinical studies in Sweden. J Neurooncol.

[51] Nakagawa Y, Pooh K, Kobayashi T (2003). Clinical review of the Japanese experience with boron neutron capture therapy and a proposed strategy using epithermal neutron beams. J Neurooncol.

[52] Miyatake S, Kawabata S, Kajimoto Y (2005). Modified boron neutron capture therapy for malignant gliomas performed using epithermal neutron and two boron compounds with different accumulation mechanisms: an efficacy study based on findings on neuroimages. J Neurosurg.

[53] Miyatake S, Kawabata S, Yokoyama K (2009). Survival benefit of boron neutron capture therapy for recurrent malignant gliomas. J Neurooncol.

[54] Kankaanranta L, Saarilahti K, Makitie A (2011). Boron neutron capture therapy (bnct) followed by intensity modulated chemoradiotherapy as primary treatment of large head and neck cancer with intracranial involvement. Radiother Oncol.

[55] Kankaanranta L, Seppala T, Koivunoro H (2012). Boron neutron capture therapy in the treatment of locally recurred head-and-neck cancer: final analysis of a phase I/II trial. Int J Radiat Oncol Biol Phys.

[56] Ariyoshi Y, Miyatake S, Kimura Y (2007). Boron neutron capture therapy using epithermal neutrons for recurrent cancer in the oral cavity and cervical lymph node metastasis. Oncol Rep.

[57] Kimura Y, Ariyoshi Y, Miyatake S (2009). Boron neutron capture therapy for papillary cystadenocarcinoma in the upper lip: a case report. Int J Oral Maxillofac Surg.

[58] Kimura Y, Ariyoshi Y, Shimahara M (2009). Boron neutron capture therapy for recurrent oral cancer and metastasis of cervical lymph node. Appl Radiat Isot.

[59] Aihara T, Hiratsuka J, Morita N (2006). First clinical case of boron neutron capture therapy for head and neck malignancies using 18f-bpa pet. Head Neck.

[60] Kato I, Ono K, Sakurai Y (2004). Effectiveness of BNCT for recurrent head and neck malignancies. Appl Radiat Isot.

[61] Kato I, Fujita Y, Maruhashi A (2009). Effectiveness of boron neutron capture therapy for recurrent head and neck malignancies. Appl Radiat Isot.

[62] Xu D, Zhang YC, Zhou QY (2021). Boron neutron capture therapy of cancers: principles and recent research progress. Chin J Radiol Med Prot.

[63] Miyatake SI, Kawabata S, Hiramatsu R (2018). Boron capture therapy of malignant gliomas. Prog Neurol Surg.

[64] Barth RF, Zhang ZZ, Liu TA (2018). realistic appraisal of boron neutron capture therapy as a cancer treatment modality. Cancer Commun.

[65] Minoru S (2020). Boron neutron capture therapy (BNCT): a unique role in radiotherapy with a view to entering the accelerator-based BNCT era. Int J Clin Oncol.

[66] Kawabata S, Miyatake S, Kuroiwa T, Yokoyama K, Doi A (2009). Boron neutron capture therapy for newly diagnosed glioblastoma. J Radiat Res.

[67] Fuwa N, Suzuki M, Sakurai Y, Nagata K, Kinashi Y, Masunaga S (2008). Treatment results of boron neutron capture therapy using intra-arterial administration of boron compounds for recurrent head and neck cancer. Br J Radiol.

[68] Wang LW, Chen YW, Ho CY (2014). Fractionated BNCT for locally recurrent head and neck cancer: experience from a phase I/II clinical trial at Tsing Hua Open-Pool Reactor. Appl Radiat Isot.

[69] Hiratsuka J, Kamitani N, Tanaka R (2020). Long-term outcome of cutaneous melanoma patients treated with boron neutron capture therapy (BNCT). J Radiat Res.

